# Different Methods for Evaluating Microglial Activation Using Anti-Ionized Calcium-Binding Adaptor Protein-1 Immunohistochemistry in the Cuprizone Model

**DOI:** 10.3390/cells11111723

**Published:** 2022-05-24

**Authors:** Mariela Wittekindt, Hannes Kaddatz, Sarah Joost, Anna Staffeld, Yamen Bitar, Markus Kipp, Linda Frintrop

**Affiliations:** Institute of Anatomy, Rostock University Medical Center, 18057 Rostock, Germany; mariela.wittekindt@med.uni-rostock.de (M.W.); hannes.kaddatz@uni-rostock.de (H.K.); sarah.joost@med.uni-rostock.de (S.J.); anna.staffeld@med.uni-rostock.de (A.S.); yamen.bitar2@uni-rostock.de (Y.B.); markus.kipp@med.uni-rostock.de (M.K.)

**Keywords:** microglia, multiple sclerosis, IBA1, cuprizone model

## Abstract

Microglia play an important role in the pathology of various central nervous system disorders, including multiple sclerosis (MS). While different methods exist to evaluate the extent of microglia activation, comparative studies investigating the sensitivity of these methods are missing for most models. In this study, we systematically evaluated which of the three commonly used histological methods (id est, quantification of microglia density, densitometrically evaluated staining intensity, or cellular morphology based on the determination of a ramification index, all measured in anti-ionized calcium-binding adaptor protein-1 (IBA1) immunohistochemical stains) is the most sensitive method to detect subtle changes in the microglia activation status in the context of MS. To this end, we used the toxin-induced cuprizone model which allows the experimental induction of a highly reproducible demyelination in several central nervous system regions, paralleled by early microglia activation. In this study, we showed that after 3 weeks of cuprizone intoxication, all methods reveal a significant microglia activation in the white matter corpus callosum. In contrast, in the affected neocortical grey matter, the evaluation of anti-IBA1 cell morphologies was the most sensitive method to detect subtle changes of microglial activation. The results of this study provide a useful guide for future immunohistochemical evaluations in the cuprizone and other neurodegenerative models.

## 1. Introduction

Microglia represent the resident innate immune cells of the central nervous system (CNS), originating from the progenitor cells of the embryonic yolk sac [1]. These immune cells migrate into the human CNS between gestational weeks 4 and 24 [2], where they propagate throughout the brain parenchyma. In the adult brain, microglia represent an important cell population to maintain the steadiness of the internal brain environment, id est, brain homeostasis. Homeostatic tasks which are known to be executed by microglia cells include the clearance of aged myelin sheath particles [3], the physical interaction with synapses of active neurons [4], or the enhancement of synaptic activity to promote local network synchronization [5]. Of note, the modulatory function of microglia in the healthy brain is only beginning to be understood. On the other hand, microglia appear to play an important role during diverse CNS diseases, such as Parkinson’s disease [6], multiple sclerosis (MS) [7], or Alzheimer’s disease [8]. Microglia constantly screen their environment for local alterations, such as the presence of cellular debris, changes in the ion concentrations, or increased levels of inflammatory cytokines, enabling them to shift toward a pro-inflammatory state when necessary [9,10,11,12,13]. This activation phenotype is characterized by a high morphological plasticity [14].

MS is a chronic inflammatory demyelinating CNS disease causing disability in more than 2.5 million people worldwide. On the histopathological level, the brains of MS patients show perivascular inflammation, peripheral immune cell recruitment, demyelination, axonal injury, as well as astrocyte and microglia activation. Of note, demyelination is not restricted to the white matter but can as well be found in the cortical and subcortical grey matter areas [15,16]. Microglia cells are believed to regulate various key pathological processes in MS. These include the release of a number of factors toxic to oligodendrocytes and neurons, such as nitric oxide (NO) and glutamate or antigen-presentation to infiltrating lymphocytes [17]. Further, MS susceptibility genes were recently found to be more frequently associated with microglia function than that of neurons or astrocytes [18]. Additionally, numerous microglia can be found in remyelinating white matter lesions, where they express markers of anti-inflammatory cytokines and immunomodulatory molecules, presumably helping to resolve inflammation [19,20,21].

To determine the role of microglia for CNS disease development and progression, it is essential to visualize their activation status in histological sections. Several methods exist to (semi-)quantify the extent of microglia activation. First, one can estimate microglia numbers within a predefined region of interest (ROI) by either assessing the number of microglia applying stereological methods [22] or by determining their densities in thin histological sections [23,24]. Second, it is possible to quantify the staining intensity by the densitometrical evaluation of these sections. Third, one can determine the morphology of microglia cells in either thin or thick histological sections [25,26,27,28]. While other (histological) methods exist to assess the activation status of microglia cells, those three listed above are currently the most commonly used methods by the scientific community.

In this study, we systematically evaluated which of these histological methods (id est, quantification of microglia numbers, staining intensity, or cellular morphology) is most sensitive to detect subtle changes in the microglia activation status in the context of MS. To this end, we used the toxin-induced cuprizone model which allows the experimental induction of a highly reproducible demyelination in several CNS regions, paralleled by robust microglia activation in the course of cuprizone intoxication [29,30].

## 2. Materials and Methods

### 2.1. Animals

C57BL6/J mice (10 weeks of age, *n* = 20, 19–23 g) were ordered from Janvier Labs (Le Genest-Saint-Isle, France) as part of a large cohort study. Female mice were only used in this study. They were housed in cages which were changed once per week with a maximum of five mice per cage with ad libitum water and food. Further, the animals were housed under standard laboratory conditions, including 12 h light/dark cycle and controlled temperature 22 ± 2 °C. As suggested by the Federation of European Laboratory Animal Science Associations (FELASA), we implemented microbiological testing. The acclimatization phase of mice lasted for seven days previous to the start of cuprizone intoxication. The assignment of animals to the experimental groups was random. The Review Boards for the Care of Animal Subjects of the district government Mecklenburg–Western Pomerania permitted the experiments (reference number 7221.3-1-001/19).

### 2.2. Cuprizone Intoxication

Cuprizone intoxication was induced as described previously [31,32,33]. Mice were intoxicated with 0.25% cuprizone mixed into standard rodent chow for 1, 3, or 5 weeks (n = 5 per group). Therefore, cuprizone was weighed using precision scales and mechanically mixed into ground standard rodent chow, using a commercially available kitchen machine. The chow was mixed at the highest speed setting and manually agitated for 1 min and was then provided in two separate plastic petri dishes per cage. Control group mice were fed with standard rodent chow throughout the experiment. The following exclusion criteria were applied: severe weight loss (>10% within 24 h), severe behavioral deficits (decreased locomotion, convulsions, stupor), or infections. No animal met the exclusion criteria during the course of this study.

### 2.3. Tissue Preparation

For tissue preparation, animals were injected intraperitoneally with ketamine (100 mg/kg) and xylazine (10 mg/kg). Afterward, mice were transcardially perfused with phosphate-buffered saline (PBS) and 3.7% paraformaldehyde solution (pH 7.4). At Bregma −2.3 (corresponding to Franklin and Paxinos atlas [34]), we made a series of 5 µm thick coronal paraffin-embedded brain sections for immunohistochemical analyses. This region was defined as region 315 (R. 315) according to the brain atlas by Sidman et al. (http://www.hms.harvard.edu/research/brain/atlas.html, accessed on 1 October 2021).

### 2.4. Immunohistochemistry/Histochemistry and Evaluation

Previously established protocols were used to conduct immunohistochemistry [35,36]. In brief, sections were deparaffinized, rehydrated, and antigens were unmasked by heating in tris(hydroxymethyl)aminomethane/ethylenediamine tetraacetic acid (Tris/EDTA) buffer (pH 9.0). After washing in PBS, the sections were incubated for 1 h in 5% blocking serum of the species in which the secondary antibody was raised. After draining the blocking serum, the sections were incubated overnight at 4 °C with the primary antibody anti-IBA1 diluted in blocking solution with a concentration of 1:500 (Wako, Neuss, Germany; 019-19741, polyclonal, RRID: AB_839504). Appropriate negative controls (omission of the primary antibody) were performed in parallel. The next day, the slides were treated with 0.35% hydrogen peroxide (H_2_O_2_) in PBS for 30 min. After washing in PBS, the slides were incubated with the EnVision System for 1 h (EnVision System-HRP labelled polymer anti-rabbit; Dako, Hamburg, Germany). The antigenic sites were detected by a reaction with 3,3′-diaminobenzidine (Dako, Hamburg, Germany) and H_2_O_2_, yielding a brownish deposit.

**Figure 1:**
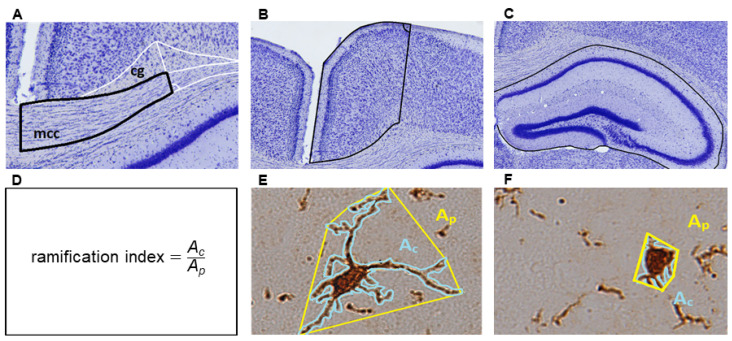
Regions of interest at brain level region 315 (Bregma −2.3; Nissl staining). (**A**) The medial corpus callosum (mcc) was defined as the corpus callosum area between a perpendicular line dropped from the interhemispheric fissure and a perpendicular line dropped from the peak of the cingulum (cg). (**B**) The medial retrosplenial cortex was defined as the cortical areas above the mcc with a perpendicular line dropped from the brain surface and the peak of the cingulum as lateral boundary. (**C**) The entire hippocampus was defined as a third region of interest (ROI). (**D**) The ramification index was calculated from the ratio of the cell area (A_c_) and the projection area (A_p_). (**E**) A_p_ is the area of a polygonal object that is defined by the cells’ most prominent projections. A resting microglia cell displays long and thin ramified processes with a relatively high A_p_ (encircled in yellow) and a relatively small A_c_ (encircled in blue). (**F**) An activated microglia cell is characterized by a hypertrophic cell body with retracted processes resulting in a ramification index value close to 1.

Figure 1 shows the ROIs (the medial corpus callosum, the medial retrosplenial cortex, and the entire hippocampus) in which the different parameters of microglia activation (quantification of microglia numbers, staining intensity, and cellular morphology) were evaluated. The medial corpus callosum was defined as the region between a perpendicular line dropped from the interhemispheric fissure and a perpendicular line dropped from the peak of the cingulum (see Figure 1A). To analyze the medial retrosplenial cortex (laminae 1–6), a perpendicular line between the peak of the cingulum and the brain surface was used as lateral boundary. Furthermore, the entire hippocampus was defined as a third ROI.

To quantify microglia cell numbers, sections were digitalized using a Nikon ECLIPSE E200 microscope (Nikon Instruments, Düsseldorf, Germany, 20-fold objective, numerical aperture (NA): 0.4) equipped with a camera (Basler acA1920-40uc). For densitometrical (4-fold objective, NA: 0.13) and morphological analyses (40-fold objective, NA: 0.95) of microglia cells, the Leica DM6 B automated microscope (Leica Microsystems CMS GmbH, Wetzlar, Germany) equipped with a DMC6200 camera was used and the images were taken using constant illumination settings. To quantify the number of microglia, the program ViewPoint (version 1.0.0.9628, PreciPoint, Freising, Germany) was used. The area of the ROI was outlined manually, and IBA1^+^ cells were counted within the ROI by two evaluators (MW and YB/AS) blinded to the treatment groups. Results are shown as the number of cells per mm^2^.

For staining intensity measurement, we performed a semi-automated densitometrical analysis with the software ImageJ (version 1.48v, NIH, Bethesda, MD, USA). The pictures were transformed to grayscale pictures and a threshold was set to divide every pixel of all pictures in black or white (two class system, binary conversion). Moreover, the threshold analysis functions by setting a value cutoff in which every pixel larger in comparison to the cutoff value refers to one class and every pixel smaller in comparison to the cutoff value refers to the other class. After setting a threshold, relative staining intensities were semi-quantified in binary converted images and the results were transformed into percentage area values. Results are shown as staining intensity in (%) area of the entire ROI.

To analyze the microglial morphology, *Z*-stack-multifocus images were generated with a 0.2 μm step size using the Leica DM6 B automated microscope (see Supplementary Figure S1 for a representative illustration). Using the software ImageJ, microglia cells in the corpus callosum (averaged analyzed cell number per animal ± standard deviation (SD): 22.15 ± 15.23 cells/animal), the cortex (16.35 ± 2.28 cells/animal), and the hippocampus (50.75 ± 18.31 cells/animal) were randomly selected by superimposing a rectangular grid with uniform distances between the lines in directions X and Y and analyzing all cells crossing the grid lines. The ramification index was calculated as illustrated in Figure 1D–F). According to Kogel et al., the ramification index is defined as the ratio of the cell area (A_c_) and the projection area (A_p_) [37]. To measure A_c_, the anti-IBA1-positive area of one individual cell was outlined using the polygon tool in ImageJ. To measure A_p_, a Convex Hull Analysis was performed which measures the size of the cellular field by interpreting a branched structure as a solid object controlling a given amount of physical space. Resting microglia are characterized by long and thin ramified processes with a relatively large A_p_ and a relatively small A_c_. Activated microglia show a hypertrophic cell body with hypertrophic and retracted cell processes. During microglial activation, the ramification index approaches a value close to 1.

### 2.5. Statistical Analyses

All data are given as arithmetic means ± SD. Differences between groups were statistically tested using the software GraphPad Prism (version 8.0.0, GraphPad Software, San Diego, CA, USA) with confidence intervals of 0.05. *p*-values of ≤ 0.05 were considered to be statistically significant. The following symbols are used to indicate the level of significance: * *p*  ≤  0.05, ** *p*  ≤  0.01, *** *p*  ≤  0.001, n.s. indicates “not significant”. Significant outliers were excluded from the densitometrical analyses using the Grubbs’ test (alpha = 0.05). To analyze differences between groups, the non-parametric Kruskal–Wallis test followed by Dunn’s multiple comparison test was applied.

## 3. Results

### 3.1. Microglia Activation in the Corpus Callosum

**Figure 2:**
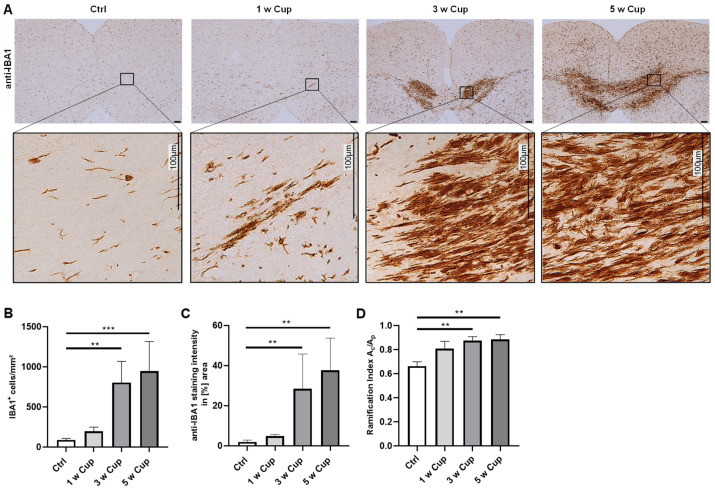
Microglia activation in the medial corpus callosum of cuprizone-intoxicated mice (n = 5 per group). C57BL/6 mice were intoxicated with cuprizone for 1, 3, or 5 weeks (1 w Cup; 3 w Cup; 5 w Cup). Controls (Ctrl) were fed with normal chow throughout the experiment. (**A**) Representative anti-IBA1 staining of Ctrl, 1, 3, and 5 w Cup mice. Insets depict representative IBA1^+^ cells in the corpus callosum (panels beneath). (**B**) IBA1^+^ cell numbers were manually counted by two independent evaluators (MW and YB) blinded to the treatment groups. The microglia cell numbers gradually increased during the course of cuprizone intoxication. (**C**) Anti-IBA1^+^ staining intensities evaluated by densitometrical analysis also increased during the course of cuprizone intoxication. Densitometrically measured IBA1^+^ staining intensities were increased in 3 and 5 w Cup mice compared to Ctrl mice. (**D**) Ramification indices of randomly selected microglia cells in the corpus callosum were determined. After 3 and 5 w of cuprizone intoxication, the ramification indices were increased compared to Ctrl mice. Kruskal–Wallis test followed by Dunn’s multiple comparisons test were used to test for significant differences between cuprizone-intoxicated and Ctrl groups. A_c_: cell area. A_p_: projection area. ** *p* ≤ 0.01, *** *p* ≤ 0.001.

In the cuprizone model, various regions of the brain are affected such as the corpus callosum which is an integral commissural fiber tract connecting the left and right cerebral hemispheres, enabling communication between them. In a first step, we analyzed the progression of microglia activation in the medial corpus callosum using the (i) quantification of anti-ionized calcium-binding adaptor protein-1 (IBA1)^+^ cell numbers, (ii) densitometrical analyses of IBA1 staining intensities, and (iii) morphological changes of IBA1^+^ cells by determining the cell ramification index (Figure 2). None of the three applied analysis methods revealed a significant increase in the analyzed parameters after 1 week cuprizone intoxication (cell number: control (Ctrl) 87.79 ± 20.52 cells/mm^2^ vs. 1 week (1 w) 196.97 ± 51.14 cells/mm^2^, n.s.; staining intensity: (Ctrl) 1.89 ± 0.96% vs. (1 w) 4.84 ± 0.84%, n.s.; ramification indices: (Ctrl) 0.66 ± 0.04 vs. (1 w) 0.81 ± 0.06, n.s.). During the course of cuprizone-induced demyelination, all three values steadily increased. At week 3, cell numbers were (Ctrl) 87.79 ± 20.52 cells/mm^2^ vs. 3 weeks (w) 803.14 ± 264.54 cells/mm^2^, *p* = 0.006; staining intensities were (Ctrl) 1.89 ± 0.96% vs. (3 w) 28.41 ± 17.31%, *p* = 0.004; and the cell ramification indices were (Ctrl) 0.66 ± 0.04 vs. (3 w) 0.87 ± 0.03, *p* = 0.008. At week 5, cell numbers were (Ctrl) 87.79 ± 20.52 cells/mm^2^ vs. (5 w) 945.12 ± 370.25 cells/mm^2^, *p* = 0.001; staining intensities were (Ctrl) 1.89 ± 0.96% vs. (5 w) 37.57 ± 16.1%, *p* = 0.002; and the cell ramification indices were (Ctrl) 0.66 ± 0.04 vs. (5 w) 0.88 ± 0.04, *p* = 0.002.

### 3.2. Microglia Activation in the Retrosplenial Cortex

**Figure 3:**
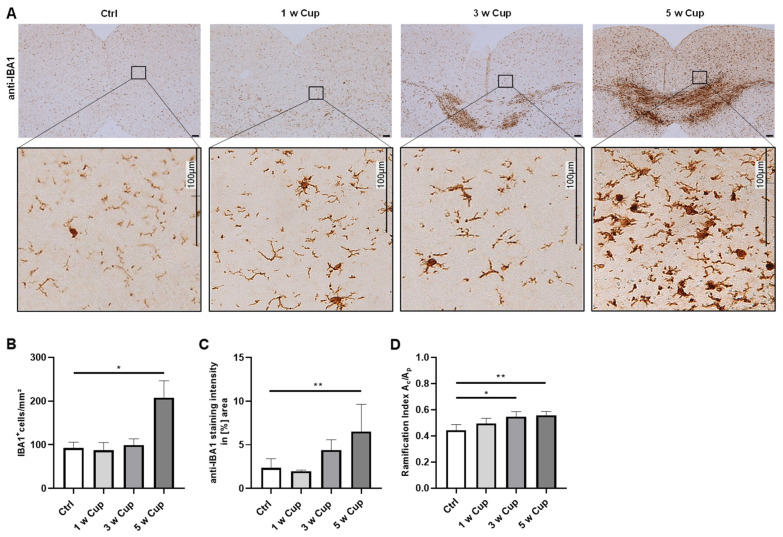
Microglia activation in the medial retrosplenial cortex of cuprizone-intoxicated mice (n = 5 per group). (**A**) Representative anti-IBA1 staining of control (Ctrl), 1, 3, and 5 w Cup mice. Insets depict representative IBA1^+^ cells in the cortex. (**B**) IBA1^+^ cell numbers were manually counted by two independent evaluators (MW and AS) blinded to the treatment groups. The densities of IBA1^+^ microglia cells were significantly increased after 5 w of cuprizone intoxication. (**C**) Densitometrically measured IBA1^+^ staining intensities were increased in 5 w Cup mice compared to Ctrl mice. (**D**) The ramification indices of microglia cells in the retrosplenial cortex gradually increased during the course of cuprizone intoxication. Kruskal–Wallis test followed by Dunn’s multiple comparisons test were used to test for significant differences between Ctrl and all cuprizone-intoxicated groups. A_c_: cell area. A_p_: projection area. * *p* ≤ 0.05, ** *p* ≤ 0.01.

During the course of cuprizone intoxication, besides the white matter tract corpus callosum, various grey matter areas, such as the neocortex, are affected as well. Due to the a priori lower myelin content in the grey matter compared to the white matter, the extent of microglia activation during the course of cuprizone-induced demyelination is less severe [38]. In a next step, we repeated the very same analyses of microglia reactivity in the retrosplenial cortical area (Figure 3). None of the methods revealed a significant change of the investigated parameters after 1 week cuprizone intoxication (cell numbers: (Ctrl) 92.31 ± 13.46 cells/mm^2^ vs. (1 w) 87.27 ± 17.79 cells/mm^2^, n.s.; staining intensity: 2.34 ± 1.07% vs. 1.97 ± 0.13%, n.s.; ramification indices: 0.44 ± 0.04 vs. 0.49 ± 0.04, n.s.). Comparable to what we observed in the corpus callosum, during the course of cuprizone-induced demyelination, all three values increased, although in a delayed time course. At week 3, cell numbers were (Ctrl): 92.31 ± 13.46 cells/mm^2^ vs. (3 w) 99.13 ± 14.54 cells/mm^2^, n.s; staining intensities were (Ctrl) 2.34 ± 1.07% vs. (3 w) 4.39 ± 1.18%, n.s.; and the cell ramification indices were (Ctrl) 0.44 ± 0.04 vs. (3 w) 0.55 ± 0.04, *p* = 0.02. At week 5, cell numbers were (Ctrl) 92.31 ± 13.46 cells/mm^2^ vs. (5 w) 207.32 ± 39.2 cells/mm^2^, *p* = 0.03; staining intensities were (Ctrl) 2.34 ± 1.07% vs. (5 w) 6.52 ± 3.12%, *p* = 0.01; and the cell ramification indices were (Ctrl) 0.44 ± 0.04 vs. (5 w) 0.56 ± 0.03, *p* = 0.005. To conclude, just the ramification index was able to detect significant microglia activation at week 3.

### 3.3. Microglia Activation in the Hippocampus Formation

**Figure 4:**
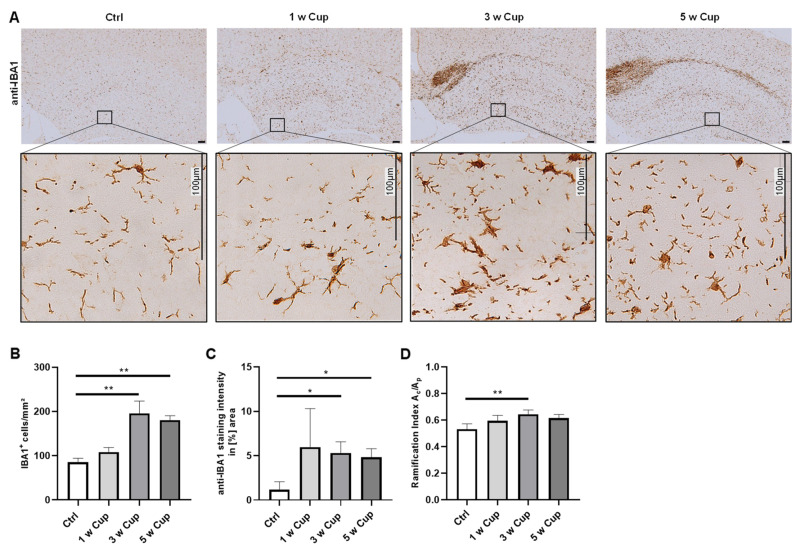
Microglia activation in the hippocampus of cuprizone-intoxicated mice (n = 5 per group). (**A**) Representative anti-IBA1 staining of control (Ctrl), 1, 3, and 5 w Cup mice. Insets depict representative IBA1^+^ cells in the hippocampus. (**B**) IBA1^+^ cell numbers were manually counted by two independent evaluators (MW and YB) blinded to the treatment groups. IBA1^+^ cell densities were significantly increased after 3 and 5 w of cuprizone intoxication. (**C**) Densitometrically measured IBA1^+^ staining intensities demonstrated a significant increase during the course of cuprizone intoxication. (**D**) The ramification indices of microglia in the hippocampus were increased after 3 w of cuprizone intoxication. Kruskal–Wallis test followed by Dunn’s multiple comparisons test were used to test for significant differences between Ctrl and all cuprizone-intoxicated groups. A_c_*:* cell area. A_p_*:* projection area. * *p* ≤ 0.05, ** *p* ≤ 0.01.

Finally, we tested by which method microglia activation in the hippocampus can be optimally analyzed (Figure 4). No method detected microglia activation at week 1 (cell number: (Ctrl) 85.42 ± 8.89 cells/mm^2^ vs. (1 w) 107.88 ± 10.77 cells/mm^2^, n.s.; staining intensity: (Ctrl) 1.17 ± 0.89% vs. (1 w) 5.94 ± 4.37% n.s.; ramification indices: (Ctrl) 0.53 ± 0.04 vs. (1 w) 0.6 ± 0.04, n.s.). Again, all three parameters increased during the course of cuprizone intoxication, but a plateau was reached at week 3 with no further increase until week 5. At week 3, cell numbers were (Ctrl) 85.42 ± 8.89 cells/mm^2^ vs. (3 w) 195.61 ± 27.98 cells/mm^2^, *p* = 0.0013; staining intensities were (Ctrl) 1.17 ± 0.89% vs. (3 w) 5.3 ± 1.26%, *p* = 0.014; and the cell ramification indices were (Ctrl) 0.53 ± 0.04 vs. (3 w) 0.64 ± 0.03, *p* = 0.003. At week 5, cell numbers were (Ctrl) 85.42 ± 8.89 cells/mm^2^ vs. (5 w) 180.56 ± 9.9 cells/mm^2^, *p* = 0.007; staining intensities were (Ctrl) 1.17 ± 0.89% vs. (5 w) 4.8 ± 0.99%, *p* = 0.05; and the cell ramification indices were (Ctrl) 0.53 ± 0.04 vs. (5 w) 0.62 ± 0.03, n.s.

## 4. Discussion

In this study, we were able to demonstrate that all of the tested methods to characterize the extent of microglia cell activation in the cuprizone model of demyelination, id est, the estimation of cell numbers, the analyses of anti-IBA1 staining intensity, and the quantification of the cellular morphology by determining the cell ramification index, can be used and provide comparable results.

In the cuprizone model, the kinetics of cellular alterations are relatively well characterized. Days after the initiation of the cuprizone intoxication, apoptotic oligodendrocytes can be histologically observed [16,39,40,41]. Oligodendrocyte stress is closely paralleled by the activation of microglia cells, as demonstrated in several studies [16,42,43]. Early microglia cell activation in this model is paralleled by the expression induction of various chemokines, including *Cxcl10*, *Ccl2*, and *Ccl3*. Some of these, for example, CXCL10, appear to be functionally involved in the complex process of microglia activation in this animal model [44,45,46,47]. Thus, early microglia activation can be determined by chemokine induction.

Microglia activation is a simple term describing a rather complex cellular process which includes changes in cell numbers, cellular morphology, gene expression, cytokine release, and the potential to present antigens, among others. While the necessity to apply reliable methods to quantify the extent of microglia activation is undisputed, the characterization of “activated” microglia is a challenging task for several reasons. For example, firstly, commonly used markers to label microglia cells in most instances also label peripheral monocytes that have invaded the brain parenchyma. Thus, a clear differentiation between microglial cells and peripheral macrophages is challenging. Secondly, while marker proteins have been identified which label microglia but not peripheral monocytes, for example, the transmembrane protein TMEM119 [48] or the purinergic receptor P2RY12, their expression is downregulated once microglia cells become activated [49,50,51]. Thirdly, while some marker proteins are expressed only by resting microglia cells (for example, TMEM119), some other marker proteins are exclusively expressed by activated microglia cells, such as the major histocompatibility complexes II (MHC-II) protein [48,52,53]. Of note, the MHC-II is not a specific protein in microglia as macrophages or B cells can also express MHC-II.

In this study, we used three commonly accepted methods to evaluate the extent of microglia cell activation, and each of the three methods has its weaknesses and strengths. Firstly, we used the parameter “number of cells”. One requirement for this method is to reliably identify the cell bodies to be able to estimate cellular numbers. This identification was challenging in the corpus callosum at week 3 and 5 of the cuprizone intoxication because of pronounced microgliosis. Further, it is worth mentioning that following the described approach, one does not evaluate cell numbers but cell densities (cells per area). To obtain entire cell numbers in a given region of interest, design-based stereology methods have to be applied as published in several models, including MS animal models [54,55]. The estimation of cell densities is a commonly used approach [56,57] and technically easy to conduct. It can preclude, at least to some extent, the interpretational error resulting from the variation in staining intensities due to differences in the tissue status and/or staining effectiveness, especially if the stains are performed manually. However, this method might underestimate the extent of microglia activation. As IBA1 protein expression is found in relevant amounts in resting microglia, minor microglia activation might not be detected by cell counting (false negative results are obtained). On the other hand, antibodies directed against marker proteins which are relatively low and expressed under physiological conditions, such as MHC-II in resting microglia [58], will reveal false positive results in injured tissues (demonstrating higher cell numbers does not necessarily mean that more microglia cells are present).

Secondly, we used the parameter “staining intensity”. The advantage of this method is that it is objective, reproducible, and can be used in areas of intense microglia activation where other methods are not precise. For example, after 5 weeks of cuprizone intoxication, the midline of the corpus callosum displays such an intense anti-IBA1 staining that cell counting and the estimation of microglia cell morphologies is complicated (compare Figure 2). A limitation of densitometrical analyses is that unspecific background staining of the investigated tissues might interfere with the evaluation of staining intensities and might, thus, bias the results. Commonly used detection systems in immunohistochemistry are, among others, the avidin–biotin complex method, the labeled streptavidin biotin method, polymer-based detection systems, or tyramine amplification systems. In comparison to standard immunohistochemical methods, polymeric and tyramine-based amplification methods have an increased sensitivity which might result in increased unspecific background signals. The choice of the detection system for immunohistochemical stains should therefore be based on the intended evaluation method. Further, appropriate threshold algorithms should be used during the digital evaluation of the staining intensity.

Finally, we determined the degree of the cellular ramification to estimate the extent of the microglia activation in anti-IBA1-stained sections. It has been demonstrated that IBA-1 regulates actin-crosslinking involved in membrane ruffling in microglia [59]. Because membrane ruffling is essential for the morphological changes occurring during microglia activation, this activation is associated with increased IBA-1 expression. Moreover, it is assumed that microglia cells play an important role in demyelination and remyelination in the course of MS, indicating that they can fulfil deleterious and beneficial functions [7,60]. To determine the role of microglia in CNS disease development, the microglia morphology have to be distinguished between an amoeboid form characterized by a hypertrophic cell body with retracted processes and a ramified cell. Further, we have to take into account that intermediate forms between amoeboid and ramified microglia also exist which might have different functions [61]. In the past years, several methods have been developed that involve morphological characteristics, such as morphological complexity with fractal [62], Skeleton [63,64], and/or Sholl analyses [65]. Roufagalas et al. recently demonstrated region-dependent alterations in microglial cell morphology in the grey matter of cuprizone-intoxicated animals analyzed with a semi-automated imaging analysis tool, MicroApp [66]. In this study, we used a relatively simple and easy-to-conduct approach, id est, an estimation of microglia ramification (see Figure 1). Ramified, resting microglial cells have a large maximum A_p_ and a relatively small A_c_. In contrast, in activated microglia cells (or macrophages), the ramification indices value approaches 1 due to a hypertrophy of the cell body paralleled by a retraction of the cell processes [26]. At least for the neocortical region, we were able to demonstrate in this study that at week 3 of the cuprizone intoxication, microglia cell morphology was significantly altered, whereas the other two parameters (cell density and staining intensity) were not yet increased. We speculate that in the white matter corpus callosum, the morphology of microglia cells is strongly related to the orientation of axonal fibers with processes running in parallel to them. Indeed, the RI values in the control corpus callosum were 0.66 (±0.04), whereas in the cortex, the RI values were profoundly lower (0.44 ± 0.04). Thus, the microglia located in the neocortical grey matter might be able to adopt their morphology faster in comparison to white matter microglia cells which would explain our observation. In line with previous results [67], the determination of cellular morphologies is an elegant and sensitive tool to estimate subtle differences in microglia cell activation in this model, at least for the neocortex.

To recommend an evaluation method of choice during the course of cuprizone intoxication, areas with intense microglia activation have to be distinguished from areas with subtle microglia changes. The cell number evaluation and the morphological method should be preferred in areas with bland microglia activation, whereas in areas with a high degree of microglia activation, densitometrical analysis appears to have a higher validity.

## Data Availability

The data that support the findings of this study are available from the corresponding author upon reasonable request.

## Supplementary Materials

The following supporting information can be downloaded at: https://www.mdpi.com/article/10.3390/cells11111723/s1, Figure S1: Representative IBA1^+^ microglia cells in Z-stack steps and multifocus images. (A) resting microglia cell in Z-stack steps and multifocus images, (B) activated microglia cell in Z-stack and multifocus images.

## References

[1] Ginhoux F., Greter M., Leboeuf M., Nandi S., See P., Gokhan S., Mehler M.F., Conway S.J., Ng L.G., Stanley E.R. (2010). Fate mapping analysis reveals that adult microglia derive from primitive macrophages. Science.

[2] Menassa D.A., Gomez-Nicola D. (2018). Microglial Dynamics During Human Brain Development. Front. Immunol..

[3] Safaiyan S., Kannaiyan N., Snaidero N., Brioschi S., Biber K., Yona S., Edinger A.L., Jung S., Rossner M.J., Simons M. (2016). Age-related myelin degradation burdens the clearance function of microglia during aging. Nat. Neurosci..

[4] Wake H., Moorhouse A.J., Jinno S., Kohsaka S., Nabekura J. (2009). Resting microglia directly monitor the functional state of synapses in vivo and determine the fate of ischemic terminals. J. Neurosci..

[5] Akiyoshi R., Wake H., Kato D., Horiuchi H., Ono R., Ikegami A., Haruwaka K., Omori T., Tachibana Y., Moorhouse A.J. (2018). Microglia Enhance Synapse Activity to Promote Local Network Synchronization. eNeuro.

[6] Ho M.S. (2019). Microglia in Parkinson’s Disease. Adv Exp. Med. Biol.

[7] Guerrero B.L., Sicotte N.L. (2020). Microglia in Multiple Sclerosis: Friend or Foe?. Front. Immunol..

[8] Hansen D.V., Hanson J.E., Sheng M. (2018). Microglia in Alzheimer’s disease. J. Cell Biol..

[9] Bogie J.F., Stinissen P., Hendriks J.J. (2014). Macrophage subsets and microglia in multiple sclerosis. Acta Neuropathol..

[10] Lassmann H. (2020). Pathology of inflammatory diseases of the nervous system: Human disease versus animal models. Glia.

[11] Czlonkowska A., Kurkowska-Jastrzebska I. (2011). Inflammation and gliosis in neurological diseases—Clinical implications. J. Neuroimmunol..

[12] Bachiller S., Jimenez-Ferrer I., Paulus A., Yang Y., Swanberg M., Deierborg T., Boza-Serrano A. (2018). Microglia in Neurological Diseases: A Road Map to Brain-Disease Dependent-Inflammatory Response. Front. Cell Neurosci..

[13] Lively S., Schlichter L.C. (2018). Microglia Responses to Pro-inflammatory Stimuli (LPS, IFNgamma+TNFalpha) and Reprogramming by Resolving Cytokines (IL-4, IL-10). Front. Cell Neurosci..

[14] Mosser D.M., Edwards J.P. (2008). Exploring the full spectrum of macrophage activation. Nat. Rev. Immunol.

[15] Di Filippo M., Portaccio E., Mancini A., Calabresi P. (2018). Multiple sclerosis and cognition: Synaptic failure and network dysfunction. Nat. Rev. Neurosci..

[16] Kipp M., Norkus A., Krauspe B., Clarner T., Berger K., van der Valk P., Amor S., Beyer C. (2011). The hippocampal fimbria of cuprizone-treated animals as a structure for studying neuroprotection in multiple sclerosis. Inflamm. Res..

[17] Dong Y., Yong V.W. (2019). When encephalitogenic T cells collaborate with microglia in multiple sclerosis. Nat. Rev. Neurol..

[18] International Multiple Sclerosis Genetics Consortium (2019). Multiple sclerosis genomic map implicates peripheral immune cells and microglia in susceptibility. Science.

[19] Lloyd A.F., Miron V.E. (2019). The pro-remyelination properties of microglia in the central nervous system. Nat. Rev. Neurol..

[20] Cignarella F., Filipello F., Bollman B., Cantoni C., Locca A., Mikesell R., Manis M., Ibrahim A., Deng L., Benitez B.A. (2020). TREM2 activation on microglia promotes myelin debris clearance and remyelination in a model of multiple sclerosis. Acta Neuropathol..

[21] Berghoff S.A., Spieth L., Sun T., Hosang L., Schlaphoff L., Depp C., Düking T., Winchenbach J., Neuber J., Ewers D. (2021). Microglia facilitate repair of demyelinated lesions via post-squalene sterol synthesis. Nat. Neurosci..

[22] Perkins A.E., Piazza M.K., Deak T. (2018). Stereological Analysis of Microglia in Aged Male and Female Fischer 344 Rats in Socially Relevant Brain Regions. Neuroscience.

[23] Norkute A., Hieble A., Braun A., Johann S., Clarner T., Baumgartner W., Beyer C., Kipp M. (2009). Cuprizone treatment induces demyelination and astrocytosis in the mouse hippocampus. J. Neurosci. Res..

[24] Hiremath M.M., Saito Y., Knapp G.W., Ting J.P., Suzuki K., Matsushima G.K. (1998). Microglial/macrophage accumulation during cuprizone-induced demyelination in C57BL/6 mice. J. Neuroimmunol..

[25] García-Magro N., Martin Y.B., Palomino-Antolin A., Egea J., Negredo P., Avendaño C. (2019). Multiple Morphometric Assessment of Microglial Cells in Deafferented Spinal Trigeminal Nucleus. Front. Neuroanat..

[26] Becker B., Demirbas M., Johann S., Zendedel A., Beyer C., Clusmann H., Haas S.J., Wree A., Tan S.K.H., Kipp M. (2018). Effect of Intrastriatal 6-OHDA Lesions on Extrastriatal Brain Structures in the Mouse. Mol. Neurobiol..

[27] Hovens I.B., Nyakas C., Schoemaker R.G. (2014). A novel method for evaluating microglial activation using ionized calcium-binding adaptor protein-1 staining: Cell body to cell size ratio. Neuroimmunol. Neuroinflamm..

[28] Davis B.M., Salinas-Navarro M., Cordeiro M.F., Moons L., De Groef L. (2017). Characterizing microglia activation: A spatial statistics approach to maximize information extraction. Sci. Rep..

[29] Goldberg J., Clarner T., Beyer C., Kipp M. (2015). Anatomical Distribution of Cuprizone-Induced Lesions in C57BL6 Mice. J. Mol. Neurosci. MN.

[30] Klein B., Mrowetz H., Barker C.M., Lange S., Rivera F.J., Aigner L. (2018). Age Influences Microglial Activation After Cuprizone-Induced Demyelination. Front. Aging Neurosci..

[31] Hochstrasser T., Exner G.L., Nyamoya S., Schmitz C., Kipp M. (2017). Cuprizone-Containing Pellets Are Less Potent to Induce Consistent Demyelination in the Corpus Callosum of C57BL/6 Mice. J. Mol. Neurosci. MN.

[32] Slowik A., Schmidt T., Beyer C., Amor S., Clarner T., Kipp M. (2015). The sphingosine 1-phosphate receptor agonist FTY720 is neuroprotective after cuprizone-induced CNS demyelination. Br. J. Pharmacol..

[33] Kaddatz H., Joost S., Nedelcu J., Chrzanowski U., Schmitz C., Gingele S., Gudi V., Stangel M., Zhan J., Santrau E. (2021). Cuprizone-induced demyelination triggers a CD8-pronounced T cell recruitment. Glia.

[34] Paxinos G., Franklin K.B.J. (2004). The Mouse Brain in Stereotaxic Coordinates.

[35] Yakimov V., Schweiger F., Zhan J., Behrangi N., Horn A., Schmitz C., Hochstrasser T., Kipp M. (2019). Continuous cuprizone intoxication allows active experimental autoimmune encephalomyelitis induction in C57BL/6 mice. Histochem. Cell Biol..

[36] Zhan J., Yakimov V., Ruhling S., Fischbach F., Nikolova E., Joost S., Kaddatz H., Greiner T., Frenz J., Holzmann C. (2019). High Speed Ventral Plane Videography as a Convenient Tool to Quantify Motor Deficits during Pre-Clinical Experimental Autoimmune Encephalomyelitis. Cells.

[37] Kogel V., Trinh S., Gasterich N., Beyer C., Seitz J. (2021). Long-Term Glucose Starvation Induces Inflammatory Responses and Phenotype Switch in Primary Cortical Rat Astrocytes. J. Mol. Neurosci..

[38] Clarner T., Diederichs F., Berger K., Denecke B., Gan L., van der Valk P., Beyer C., Amor S., Kipp M. (2012). Myelin debris regulates inflammatory responses in an experimental demyelination animal model and multiple sclerosis lesions. Glia.

[39] Mason J.L., Jones J.J., Taniike M., Morell P., Suzuki K., Matsushima G.K. (2000). Mature oligodendrocyte apoptosis precedes IGF-1 production and oligodendrocyte progenitor accumulation and differentiation during demyelination/remyelination. J. Neurosci. Res..

[40] Goldberg J., Daniel M., van Heuvel Y., Victor M., Beyer C., Clarner T., Kipp M. (2013). Short-term cuprizone feeding induces selective amino acid deprivation with concomitant activation of an integrated stress response in oligodendrocytes. Cell. Mol. Neurobiol..

[41] Buschmann J.P., Berger K., Awad H., Clarner T., Beyer C., Kipp M. (2012). Inflammatory response and chemokine expression in the white matter corpus callosum and gray matter cortex region during cuprizone-induced demyelination. J. Mol. Neurosci. MN.

[42] Plant S.R., Wang Y., Vasseur S., Thrash J.C., McMahon E.J., Bergstralh D.T., Arnett H.A., Miller S.D., Carson M.J., Iovanna J.L. (2006). Upregulation of the stress-associated gene p8 in mouse models of demyelination and in multiple sclerosis tissues. Glia.

[43] Hoyos H.C., Rinaldi M., Mendez-Huergo S.P., Marder M., Rabinovich G.A., Pasquini J.M., Pasquini L.A. (2014). Galectin-3 controls the response of microglial cells to limit cuprizone-induced demyelination. Neurobiol. Dis..

[44] Clarner T., Janssen K., Nellessen L., Stangel M., Skripuletz T., Krauspe B., Hess F.M., Denecke B., Beutner C., Linnartz-Gerlach B. (2015). CXCL10 triggers early microglial activation in the cuprizone model. J. Immunol..

[45] Kramann N., Menken L., Pförtner R., Schmid S.N., Stadelmann C., Wegner C., Brück W. (2019). Glial fibrillary acidic protein expression alters astrocytic chemokine release and protects mice from cuprizone-induced demyelination. Glia.

[46] Krauthausen M., Saxe S., Zimmermann J., Emrich M., Heneka M.T., Müller M. (2014). CXCR3 modulates glial accumulation and activation in cuprizone-induced demyelination of the central nervous system. J. Neuroinflamm..

[47] Skripuletz T., Hackstette D., Bauer K., Gudi V., Pul R., Voss E., Berger K., Kipp M., Baumgärtner W., Stangel M. (2013). Astrocytes regulate myelin clearance through recruitment of microglia during cuprizone-induced demyelination. Brain J. Neurol..

[48] Vankriekelsvenne E., Chrzanowski U., Manzhula K., Greiner T., Wree A., Hawlitschka A., Llovera G., Zhan J., Joost S., Schmitz C. (2022). Transmembrane protein 119 is neither a specific nor a reliable marker for microglia. Glia.

[49] Zrzavy T., Hametner S., Wimmer I., Butovsky O., Weiner H.L., Lassmann H. (2017). Loss of ‘homeostatic’ microglia and patterns of their activation in active multiple sclerosis. Brain J. Neurol..

[50] Marzan D.E., Brügger-Verdon V., West B.L., Liddelow S., Samanta J., Salzer J.L. (2021). Activated microglia drive demyelination via CSF1R signaling. Glia.

[51] Deczkowska A., Keren-Shaul H., Weiner A., Colonna M., Schwartz M., Amit I. (2018). Disease-Associated Microglia: A Universal Immune Sensor of Neurodegeneration. Cell.

[52] Hayes G.M., Woodroofe M.N., Cuzner M.L. (1987). Microglia are the major cell type expressing MHC class II in human white matter. J. Neurol. Sci..

[53] Wolf Y., Shemer A., Levy-Efrati L., Gross M., Kim J.S., Engel A., David E., Chappell-Maor L., Grozovski J., Rotkopf R. (2018). Microglial MHC class II is dispensable for experimental autoimmune encephalomyelitis and cuprizone-induced demyelination. Eur. J. Immunol..

[54] Vogt J., Paul F., Aktas O., Müller-Wielsch K., Dörr J., Dörr S., Bharathi B.S., Glumm R., Schmitz C., Steinbusch H. (2009). Lower motor neuron loss in multiple sclerosis and experimental autoimmune encephalomyelitis. Ann. Neurol..

[55] Kipp M., Kiessling M.C., Hochstrasser T., Roggenkamp C., Schmitz C. (2017). Design-Based Stereology for Evaluation of Histological Parameters. J. Mol. Neurosci. MN.

[56] Mohammadi M., Abdi M., Alidadi M., Mohamed W., Zibara K., Ragerdi Kashani I. (2021). Medroxyprogesterone acetate attenuates demyelination, modulating microglia activation, in a cuprizone neurotoxic demyelinating mouse model. Am. J. Neurodegener. Dis..

[57] Wies Mancini V.S.B., Pasquini J.M., Correale J.D., Pasquini L.A. (2019). Microglial modulation through colony-stimulating factor-1 receptor inhibition attenuates demyelination. Glia.

[58] Subbarayan M.S., Hudson C., Moss L.D., Nash K.R., Bickford P.C. (2020). T cell infiltration and upregulation of MHCII in microglia leads to accelerated neuronal loss in an α-synuclein rat model of Parkinson’s disease. J. Neuroinflamm..

[59] Sasaki Y., Ohsawa K., Kanazawa H., Kohsaka S., Imai Y. (2001). Iba1 is an actin-cross-linking protein in macrophages/microglia. Biochem. Biophys. Res. Commun..

[60] Pons V., Rivest S. (2020). Beneficial Roles of Microglia and Growth Factors in MS, a Brief Review. Front. Cell Neurosci..

[61] Okajima T., Tsuruta F. (2018). Microglial dynamics during brain development. Neural Regen. Res..

[62] Karperien A., Ahammer H., Jelinek H.F. (2013). Quantitating the subtleties of microglial morphology with fractal analysis. Front. Cell Neurosci..

[63] Young K., Morrison H. (2018). Quantifying Microglia Morphology from Photomicrographs of Immunohistochemistry Prepared Tissue Using ImageJ. J. Vis. Exp..

[64] Morrison H.W., Filosa J.A. (2013). A quantitative spatiotemporal analysis of microglia morphology during ischemic stroke and reperfusion. J. Neuroinflamm..

[65] Hinwood M., Tynan R.J., Charnley J.L., Beynon S.B., Day T.A., Walker F.R. (2013). Chronic stress induced remodeling of the prefrontal cortex: Structural re-organization of microglia and the inhibitory effect of minocycline. Cereb. Cortex.

[66] Roufagalas I., Avloniti M., Fortosi A., Xingi E., Thomaidou D., Probert L., Kyrargyri V. (2021). Novel cell-based analysis reveals region-dependent changes in microglial dynamics in grey matter in a cuprizone model of demyelination. Neurobiol. Dis..

[67] Franciosi S., Ryu J.K., Shim Y., Hill A., Connolly C., Hayden M.R., McLarnon J.G., Leavitt B.R. (2012). Age-dependent neurovascular abnormalities and altered microglial morphology in the YAC128 mouse model of Huntington disease. Neurobiol. Dis..

