# Supplementary material for: Different Methods for Evaluating Microglial Activation Using Anti-Ionized Calcium-Binding Adaptor Protein-1 Immunohistochemistry in the Cuprizone Model
Source: Cells. 2022 May 24;11(11):1723. doi: 10.3390/cells11111723 (PMC9179561; doi:10.3390/cells11111723)
Supplement: Supplementary file 1 [file cells-11-01723-s001.zip › cells-1721930-supplementary.pdf]

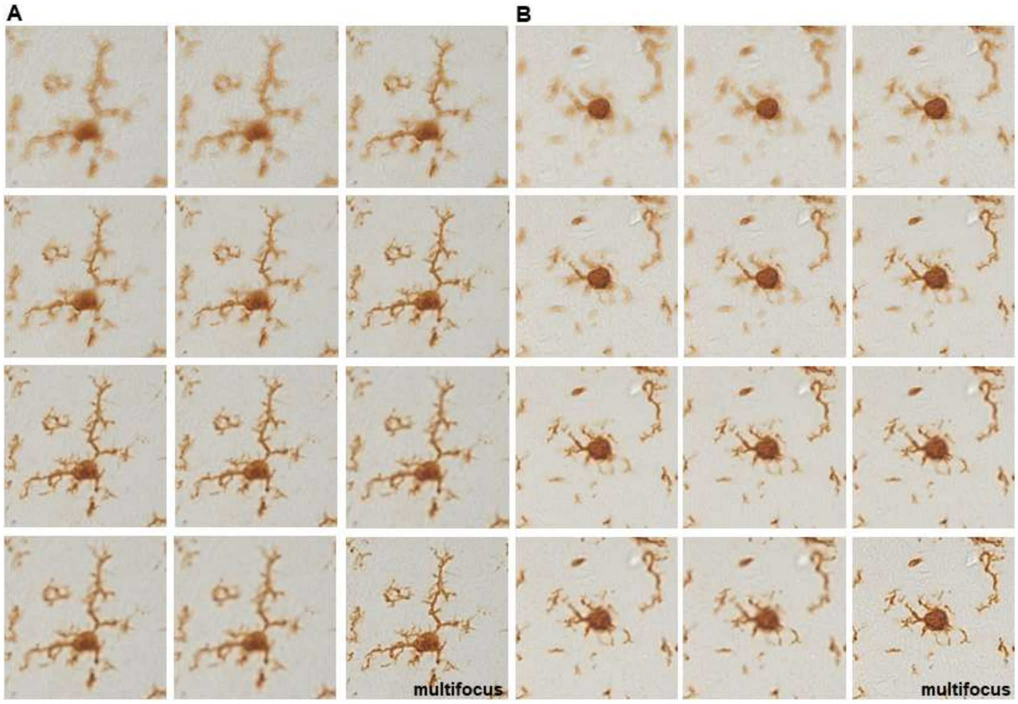

Figure S1: Representative IBA1⁺ microglia cells in Z-stack steps and multifocus images. (A) resting microglia cell in Z-stack steps and multifocus images, (B) activated microglia cell in Z-stack and multifocus images.
